# Reproductive Performance, Udder Health, and Antibiotic Resistance in Mastitis Bacteria isolated from Norwegian Red cows in Conventional and Organic Farming

**DOI:** 10.1186/1751-0147-52-11

**Published:** 2010-02-08

**Authors:** Randi T Garmo, Steinar Waage, Ståle Sviland, Britt IF Henriksen, Olav Østerås, Olav Reksen

**Affiliations:** 1 Norwegian School of Veterinary Science, Department of Production Animal Clinical Sciences, P.O. Box 8146 Dep., NO-0033 Oslo, Norway; 2 National Veterinary Institute, NO-0106 Oslo, Norway; 3 Norwegian Institute for Agricultural and Environmental research, Organic Food and Farming Division, NO-6630 Tingvoll, Norway

## Abstract

**Background:**

The objectives of this study were to investigate whether there were differences between Norwegian Red cows in conventional and organic farming with respect to reproductive performance, udder health, and antibiotic resistance in udder pathogens.

**Methods:**

Twenty-five conventional and 24 organic herds from south-east and middle Norway participated in the study. Herds were matched such that geographical location, herd size, and barn types were similar across the cohorts. All organic herds were certified as organic between 1997 and 2003. All herds were members of the Norwegian Dairy Herd Recording System. The herds were visited once during the study. The relationship between the outcomes and explanatory variables were assessed using mixed linear models.

**Results:**

There were less > 2nd parity cows in conventional farming. The conventional cows had higher milk yields and received more concentrates than organic cows. Although after adjustment for milk yield and parity, somatic cell count was lower in organic cows than conventional cows. There was a higher proportion of quarters that were dried off at the herd visit in organic herds. No differences in the interval to first AI, interval to last AI or calving interval was revealed between organic and conventional cows. There was no difference between conventional and organic cows in quarter samples positive for mastitis bacteria from the herd visit. Milk yield and parity were associated with the likelihood of at least one quarter positive for mastitis bacteria. There was few *S. aureus *isolates resistance to penicillin in both management systems. Penicillin resistance against Coagulase negative staphylococci isolated from subclinically infected quarters was 48.5% in conventional herds and 46.5% in organic herds.

**Conclusion:**

There were no large differences between reproductive performance and udder health between conventional and organic farming for Norwegian Red cows.

## Background

Organic agriculture aims to be a holistic production management system which promotes and enhances ecosystem health, including biological cycles and soil biological activity. The primary goal for organic agriculture is to optimize the health and productivity of inter-dependent communities of soil life, plants, animals and people [1]. Organic farms are supposed to be self-sufficient for animal feed, and the use of chemical fertilizers or herbicides are prohibited. In recent years, there has been increased attention to organic farming. According to recent statistics, 3.9% of the cultivated land, 2.1% of total milk production, and 2.6% (6800 cows) of the dairy cows in Norway are managed organically[2]. The legislation governing Norwegian organic farming is based on principles derived from International Federation of Organic Agriculture Movements [2]. At least 50% of feed on an organic farm should be produced on the farm itself and roughage should constitute 60% of energy fed in dry matter intake. The proportion of roughage can be reduced to 50% the first three months of lactation.

Organic regulations vary between countries, so it is virtually impossible to compare treatments and disease rates among the various systems. In Norway, the use of synthetic veterinary products prophylactically is prohibited. Withdrawal times for prescribed products are twice as long as corresponding time periods for conventional farming. During one year a maximum of three treatments periods with drugs are allowed for each individual. Natural mating is preferred over artificial insemination (AI). Embryo transfer and estrus synchronization programs are prohibited. On the individual level, hormones such as gonadotropin releasing hormone and prostaglandins are only allowed for treatment of ovarian cysts or luteolysis [3].

Lower milk yield in organic managed cows have been reported worldwide [4-10]. Impaired reproductive performance has been reported in organic cows [4]. The differences were due to a limited energy intake and increased winter breeding in the organic cows. In a Swedish study, the calving interval and the intervals from calving to first and last AI, were shorter for organic cows compared to conventional cows [11]. Nauta et al. reported an extended calving interval in cows managed on organic farms that converted to an organic management system between 1990 and 2003 [7].

Differing results have been reported for udder health when organic and conventional dairy herds were compared. A lower mastitis treatment rate has been found in organic herds, which might be due to lower milk yield in these herds [5,12,13]. However, Hardeng and Edge did not find any significant difference in individual cow milk somatic cell count (SCC) between organic and conventional herds [5], and Valle et al. found no difference in bulk milk SCC [13].

The use of antibiotics in organic farming is based on country specific regulations. In Norway three treatments periods with antibiotics are allowed for each individual during a year [3]. In Norway, Denmark, and Sweden the withdrawal period is extended after use of antibiotics to restrict the use[3,14,15]. In Switzerland and Denmark the prophylactic use of antibiotics is prohibited [14,16]. In US organic animals may not receive antibiotics if milk and meat is to be sold as organic [17-19]. The lower treatment rate and, thus, reduced use of antibiotics, may reduce antibacterial selection pressure. A few studies have been carried out comparing the occurrence of antibiotic resistant udder pathogens in organic and conventional farming [16,17,20,21]. Roesch et al. reported no difference in antibiotic resistance [16], Tikofsky et al. (2003) found good susceptibility to the most commonly used antibiotics [20], and Sato et al. (2004) reported small differences between conventional and organic farming [21]. Pol and Ruegg reported that use of penicillin was associated with reduced susceptibility of *S. aureus *and CNS isolates [17].

The relationship between selection for milk yield and reproductive performance and time to onset of luteal activity post partum has been investigated previously [22]. Cows selected exclusively for high milk yield had a longer interval to the commencement of luteal activity after calving than cows bred according to other breeding objectives. A prolonged period of ovarian quiescence was found to be reduced if selection for milk yield was combined with fertility in the breeding program. Rozzi et al. reported that organic farmers emphasized functional traits rather than production traits in Holstein cows [23]. For Norwegian Red cows, it is reported that it is possible to obtain genetic improvement for clinical mastitis and milk yield simultaneously if the traits are given sufficient weight in selection [24] and that selection against clinical mastitis is favorable correlated with selection responses for ketosis and retained placenta [25]. Since the breeding program for the Norwegian Red has paid attention to fertility, health and functional traits for a long period, this breed may perform well with regards to fertility and udder health in both conventional and organic farming systems.

The objectives of this study were to investigate whether there were differences between Norwegian Red cows managed conventionally and organically with respect to reproductive performance, udder health, and resistance against penicillin in udder pathogens.

## Methods

### Sampling of herds

The conventional and organic herds present in the selected cohort were registered in the Norwegian Dairy Herd Recording System (NDHRS). The organic farms converted at least four years before the start of the study. The organic herds were certified as organic between 1997 and 2003. All organic herds in south east (n = 26) and middle Norway (n = 21), excluding herds with less than nine cow-years (one cow-year = 365 d for a cow in the herd during one year), received an invitation letter for participation in the study. Farmers were requested to reply within one week to be included in the study. Those that did not were contacted by telephone one week after the deadline. Thirty of the 47 farms contacted agreed to participate in the study. Four of the thirty were excluded because they contained breeds other than the Norwegian Red. A further herd was excluded as it was farmer cooperatively and so had an elevated number of herdsmen. Another herd decided to not participate at the start of the study.

Thirty eight owners of conventional herds located in the same geographical areas as organic herds were asked to participate. Herds were matched with the organic farms according to herd size (± five cow-years) and type of housing. Eleven farmers were not interested (mostly due to the extra labor), one farmer planned cooperative farming, and one farmer planned conversion to organic farming.

In total, 24 organic and 25 conventional herds were enrolled in the study. One organic herd ended milk production during the study period. The distribution of geographical area, herd size, and barn type is presented in Table 1. The veterinarians practicing on the selected farms were informed about the study before it started, and were asked to collect quarter milk samples and complete a standard form when treating cases of clinical mastitis in these herds. Bacteriological examination of milk samples were performed free of charge for the farmer.

**Table 1:** Distribution of herds in organic and conventional farming.

		Organic herds		Conventional herds	
			
		n	%	n	%
Geographical area	South-east	17	70.8	18	72.0
	Middle	7	29.1	7	28.0
					
Herd size	≤ 10 cow-years	1	4.2	1	4.0
	11-20 cow-years	12	50.0	11	44.0
	21-30 cow-years	8	33.3	10	40.0
	>31 cow-years	3	12.5	3	12.0
					
Barn type	Tie stall	10	41.7	9	36.0
	Free- tall	14	58.3	16	64.0
					
Parity 2005 to 2007	1st	580	37.9	706	42.0
	2nd	381	24.9	453	27.0
	> 2nd	568	37.1	521	31.0

Distribution of the organic (n = 24) and the conventional (n = 25) dairy herds according to geographical area, herd size, barn type, and parities from 2005 to 2007.

### Individual and Herd data

Individual animal data for test-day milk yield, test-day SCC, and test-day concentrate allocation (CA), calving date, the intervals from calving to first and last AI, and use of natural mating or AI were obtained for all cows enrolled in NDHRS from 2005 throughout 2007 (n = 3209 lactations, 2093 cows). The calving interval was defined as time between two successive calvings ≤ 550 d, and days open were calculated as calving interval minus 281 d (expected gestation period). Cows with no data on AI in the NDHRS were assumed to be naturally mated 281 d prior to calving. The milk yield recording closest to 28 DIM (range 15 to 45 d, n = 845) was used as an estimate for milk yield in the fourth week of lactation. Occurrence of clinical mastitis from February 2006 to August 2007 that was treated and reported was obtained from the NDHRS files. A new clinical mastitis treatment within 9 d after the initial treatment was not considered to be a new case, because a new milk sample was not obtained from any of these cases. Cases which had been treated for retained placenta and reproductive disorders from 2005 throughout 2007 were obtained from the NDHRS files. The average annual forage: concentrate ratio for the herds and culling rate (number of culled cows per 100 cow-years) of cows in 2006 and 2007 was also obtained from the NDHRS files.

### Herd visit

Each herd was visited once by the same researcher (first author) between February and June in 2006. During this visit quarter milk samples for bacteriological examination were collected aseptically from 523 conventional and 487 organic lactating cows. Milk was collected aseptically from all quarters of cows in lactation on the respective day of the herd visit. The California mastitis test (CMT) was performed when the milk was sampled. The milk samples were refrigerated immediately after sampling. Samples that were not examined the day after sampling (29 herds) were frozen at -18°C in 2 to13 d until they underwent bacteriological examination.

### Milk samples obtained from cases of clinical mastitis

The farmers were requested to collect quarter milk samples from all cows affected by clinical mastitis or showing signs that might indicate the presence of clinical mastitis between February 2006 and August 2007. Samples were to be collected regardless of whether a veterinary surgeon was contacted. A written description of the signs of clinical mastitis (i.e., visible changes in milk such as clots, yellow like, blood like or water like milk, changes in quarters such as soreness/ache or pain by palpation), was given to the farmers. Standard packages for milk collection and transportation of quarter milk were distributed. The farmers received information about routines required for aseptic milk sampling and interpretation of CMT. Information about the condition of clinical cases was recorded on a standard form, which included the rectal temperature, appetite (recorded as normal, slightly decreased, markedly decreased, anorexia) and clinical signs of acute or chronic inflammation at quarter level, teat injury, and visual abnormality of secretion. CMT was recorded on a scale from 1 to 5 [26]. The farmers were instructed to keep the milk samples cold and submit the samples as soon as possible by mail to the National Veterinary Institute.

### Laboratory Methods

Bacteriological examinations of quarter milk samples were performed at the Mastitis Laboratory of the National Veterinary Institute, Oslo, Norway. Secretions were brought to room temperature, assessed visually and characterized by appearance. After being mechanically shaken, secretions (0.01 ml) were plated on Bacto Blood Agar Base No 2 (Difco Laboratories, Detroit, MI, USA) containing 5% washed bovine erythrocytes and incubated for 48 h in a 5% CO_2 _atmosphere at 37°C. Cultures were read at 24 and 48 h. If growth was not detected after 24 h incubation, the original sample was incubated for 4 h at 37°C and 0.05 ml was plated on blood agar and incubated for 24 h under aerobic (5% CO_2 _atmosphere) and anaerobic conditions.

Bacteria were identified according to the recommendations of the International Dairy Federation [27]. Species were identified tentatively by gross colony morphology and Gram staining; further confirmatory tests were used as necessary. Suspected staphylococcal colonies were tested using the tube coagulase test (Becton Dickinson Microbiology Systems, MA, USA). Staphylococci were differentiated from streptococci with a catalase test and *Streptococcus dysgalactiae *subspecies *dysgalactiae, Streptococcus uberis *and *Streptococcus agalactiae *were differentiated by the CAMP reaction and their ability to hydrolyze aesculin and inulin. *Escherichia coli *was identified by lactose and indole fermentation tests; other bacteria within the family *Enterobacteriaceae *were identified to species or genus level by the API 20 E^® ^identification system (bioMérieux, Marcy l'Etoile, France). Staphylococcal isolates were tested for β-lactamase activity by the cloverleaf method [28] using *Staphylococcus aureus *ATCC 25923 as the indicator strain.

### Statistical analyses

The unit of the study was lactation periods. Statistical significance was considered at *P *≤ 0.05 for all analyses.

#### Chi-square tests

Pearson Chi-square tests were performed to assess the univariate relationships between management systems and the following variables; parity distributions, proportion dried off quarters, AI versus natural mating, season for first AI, proportion of milk samples negative for pathogens, and proportion of milk samples from clinical mastitis negative for pathogens.

#### Associations between reproductive performance and management system, milk yield, season, parity, and AI versus natural mating

Mixed linear models were performed using SAS 9.1 [29] to separately assess the relationships between the three outcome variables; interval from calving to first AI, interval from calving to last AI, and calving interval ≤ 550 d and the explanatory variables; management system, parity, milk yield in the fourth week of lactation, barn type, and season for first AI. In addition, calving interval was also assessed with AI versus natural mating. The interval from calving to first AI (lnCFAI) and the interval from calving to last AI (lnCLAI) were transformed by natural logarithm to approximate normality of the residuals. Explanatory variables with *P *≤ 0.20 in the separate analyses were included in the final models together with the interactions terms; management system by parity, parity by season, management system by season, barn type by season, barn type by management system, barn type by parity. Herd was included in the models as random effect. The backward elimination procedure was applied for explanatory variables with *P *> 0.10 in the extended model. The covariance between multiple measurements of lnCFAI, lnCLAI and calving interval was correlated, and the models were adjusted for multiple lactations within the same cow by the use of a compound symmetry correlation structure for repeated effects. Multiple comparison adjustment for the pair wise difference in least square means (**LS-means**) was performed using the Bonferroni option.

#### Lactations curves for test-day milk yield, somatic cell count and concentrate allocation

For construction of the lactation curves for the continuous variables; test-day milk yield, test-day SCC and test-day CA multiple records on milk yield, SCC and CA were used for each lactation. Test-day milk yield and CA was recorded monthly whereas SCC was recorded bimonthly. Only recordings within 305 DIM were included in the analysis. Number of lactations used to construct the lactation curves from 2005 to 2007 was 3088, 3096, and 3035 for test-day milk yield, CA, and SCC, respectively. The outcome variable, SCC was transformed by natural logarithm (lnSCC) to obtain approximate normality of residuals. Mixed linear models for repeated outcomes were run using SAS 9.1[29] to assess the relationships between the outcome variables (milk yield, lnSCC, and CA) and the explanatory variables. Subjects were lactation within cows and week in milk were entered as repeated effect. Covariance between multiple measurements of milk yield, lnSCC, and CA within lactations were correlated and accounted for by the use of first order autoregressive correlation structure moving average (ARMA).

The lactation curves were expressed through inclusion of weeks in milk (WIM) and the natural logarithm of WIM (lnWIM) as described by [30-32]. The explanatory variables WIM and lnWIM were entered simultaneously in all models with test-day milk yield, lnSCC or CA as outcome variables. The explanatory variables; management system (conventional or organic) and parity (1st, 2nd, and > 2nd) were first assessed separately for each of the three outcome variables (milk yield, lnSCC, and CA). Secondly, interaction terms considered as biological important such as WIM by management, WIM by parity, lnWIM by management, and lnWIM by parity were included in the respective models. Thirdly, also test-day milk yield was included as an explanatory variable in a separate model expressing the lactation curve for lnSCC. Herd was included in the models as random effect. The final model for each of the three outcomes was constructed such that the explanatory variables and their interactions terms with *P *≤ 0.20 when tested separately, were included in an extended model. The backward elimination procedure was applied for the explanatory variables and interaction terms with *P *> 0.10 in the extended model.

#### Associations between cows with milk samples positive for mastitis bacteria and management system, milk yield, parity, and WIM

Intra-mammary infection was considered to be present when an udder pathogen was isolated from at least one quarter. Associations between the presence of intra-mammary infection (= 1) or not (= 0) at the herd visit and the explanatory variables; management, milk yield in the fourth wk of lactation, WIM, and parity were tested separately using random effects logistic model in STATA 11.0. Herd was added as random effect in the model. Explanatory variables with *P *≤ 0.20 when assessed separately and the interaction terms; parity by WIM, parity by milk yield, and WIM by milk yield were included in an extended model. The backward elimination procedure was applied for explanatory variables with *P *> 0.10 in the extended model.

## Results

### Herd characteristics

The average herd size in 2006 was 23.8 (SD ± 11.8) and 23.0 (SD ± 11.5) cow-year for the conventional and organic herds, respectively. Average milk yield per cow-year in 2006 was 7188 (SD ± 805) and 6155 kg (SD ± 963) for conventional and organic herds, respectively. The distribution of parities for conventional and organic farming from 2005 to 2007 is presented in Table 1. There were more 1st parity and less > 2nd parity cows in conventional compared to organic farming (*P *< 0.01). The culling rate for 2007 was 46.9/100 cow-year (95% CI: 35.0, 58.9) in conventional farming and 31.2/100 cow-year (95% CI: 25.3, 37.1) in organic farming. The average annual forage: concentrate ratio for conventional herds was 63:37 (95% CI: 35.1, 39.5) and 75:25 (95% CI: 22.6, 28.3) for organic herds.

There were 47 cases of retained placenta recorded in the NDHRS files from conventional herds, whereas there were 19 cases recorded from organic herds from 2005 throughout 2007. There were 148 cases of treatments for reproductive disorders (silent heat, heat synchronization, metritis, endometritis, vaginitits, cystic ovaries, and repeated breeding) in conventional herds during the same period, whereas the corresponding figures were 15 in organic herds.

### Descriptive reproductive parameters

There were 1548 and 1403 lactations with records on AI or natural mating as first service for the conventional and organic herds, respectively. Natural mating was preferred as first service in 2.8% (43/1548) of the conventional observations and in 13.8% (199/1403) of the organic observations (*P *< 0.01). Natural mating was registered in six organic and six conventional farms. There were two organic farms using mostly natural mating as first service with 100% and 73.5% matings registered. For the conventional farms, natural mating as first service was used in 5.4% to 33.3% of the cows.

There were no differences between conventional and organic cows in the overall calving interval or in the calving interval when only cows presented for first AI were included (Table 2). When only cows presented for natural mating at first service were included, the median calving interval was 341 and 382 d for organic and conventional cows, respectively. There were no differences between days open, days to first AI, days to last AI between conventional and organic cows (Table 2).

**Table 2:** Descriptive reproduction parameters.

	Conventional				Organic			
								
	n	median	mean	95% CI	n	median	mean	95% CI
Calving interval								
*Overall*	1112	367	376.3	373.5, 379.0	1102	365	375.6	372.7, 378.5
*Only AI*	1074^1^	366	375.8	373.0, 378.5	922^1^	368	379.5	376.4, 382.6
*Only natural mating*	37^1^	382	386.1^a^	372.4, 399.7	177^1^	341	355.0^b^	347.7, 362.4
								
Days open								
*Overall*	1112	86	95.3	92.5, 98.0	1102	84	94.6	91.7, 97.5
*Only AI*	1074^1^	85	94.8	92.0, 97.5	922^1^	87	98.5	95.4, 101.6
*Only natural mating*	37^1^	101	105.1^a^	91.4, 118.7	177^1^	60	74.0^b^	66.7, 81.4
								
CFAI	1505	72	79.5	77.8, 81.2	1204	76	82.0	80.2, 83.8
CLAI	1505	90	98.8	96.4, 101.2	1204	90	99.0	96.5, 101.5

^1 ^For one conventional and three organic cows that calved data was missing for breeding management and days open.

^a, b ^Different subscripts indicates significant differences between conventional and organic farming by Bonferroni adjustment for multiple comparisons, *P *< 0.05

Calving interval (overall, AI only, naturalmating only), interval from calving to first AI (CFAI), interval from calving to last AI (CLAI), and number of AI per observations in conventional and organic farming.

There was no difference between season for AI in conventional and organic farming. First AI was performed during the summer season (April-September) in 58.4% (875/1498) and 56.3% (675/1198) of the lactations in the conventional and organic farms, respectively.

### Associations between interval to first AI and management, milk yield, parity barn type, and season

When lnCFAI was assessed in separate models for each of the explanatory variables separately, *P*-values ≤ 0.20 were observed for management (*P *= 0.01), parity (*P *= 0.09), barn type (*P *≤ 0.01), and season (*P *= 0.16). After the backwards selection procedure had been applied, no explanatory variables remained to be associated with lnCFAI.

### Associations between interval to last AI and management, milk yield, parity, and season

When lnCLAI was assessed in separate models for each of the explanatory variables, *P*-values ≤ 0.20 were observed only for parity (*P *< 0.01) and barn type (*P *< 0.01). After the backwards selection procedure had been applied, both parity (*P *< 0.01) and barn type (*P *= 0.03) remained in the model. None of the interaction terms were associated with lnCLAI. The results from the extended model can be obtained in Table 3. The back transformed LS-means for the interval to last AI were 95.1, 89.7, and 93.0 d for 1st, 2nd, and > 2nd parity, respectively. The back transformed LS-means for the interval to last AI were 87.1 and 97.3 d for free stall and tie stall cows, respectively.

**Table 3:** Models for assessment of reproductive performance.

		β	SE	*P*	Ls-means
lnCLAI (n = 2709)					
Parity	1st parity^1^	-	-	-	4.555^a^
	2nd parity	- 0.059	0.019	< 0.01	4.496^b^
	> 2nd parity	- 0.023	0.019	0.24	4.533^a, b^
Barn type	Tie stall	-	-	-	4.478^a^
	Free stall	-0.100	0.043	0.02	4.578^b^
Intercept		4.605	0.035	< 0.01	
					
Calving interval (n = 2039)					
Intercept		384.80	3.723	< 0.01	-
Parity	1^st ^parity^1^	-	-	-	380.8^a^
	2^nd ^parity	-5.502	2.200	0.01	375.4^b^
	> 2^nd ^parity	-1.077	2.202	0.63	379.8^a, b^
Barn type	Tie stall	-	-	-	374.7
	Free stall	-7.897	4.492	0.07	382.6

Associations between outcomes for reproductive performance; natural logarithm of the interval to last AI (lnCLAI), and calving interval and the explanatory variables; management, season, parity, and breeding management as assessed by mixed linear models adjusted for multiple lactations within cow by the use of compound symmetry correlation structure and herd as random effect.

### Associations between calving interval and management, season, breeding management, and parity

When calving interval was assessed in the separate models for each of the explanatory variables, *P*-values ≤ 0.20 were observed for breeding management (*P *< 0.01), parity (*P *= 0.09), barn type (*P *< 0.01), and season (*P *= 0.20). After the backwards selection procedure was applied, parity (*P *= 0.04) and barn type (*P *= 0.08) remained in the model. LS-means for calving interval was 380.8, 375.4, and 379.8 d for 1st, 2nd, and > 2nd parity, respectively (Table 3).

### Test-day milk yield

**Figure 1:**
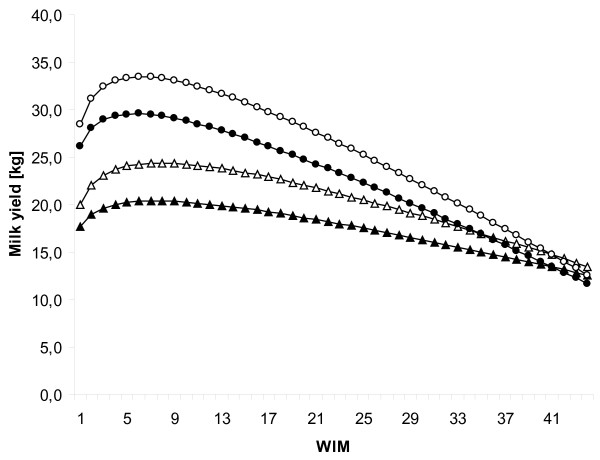
**Lactation curves for milk yield**. Predicted test-day milk yield (kg/d) by WIM for 1st (organic = black triangle, conventional = white triangle) and > 2nd (organic = black circle, conventional = white circle) parity cows in conventional and organic farming.

All explanatory variables; WIM, lnWIM, management, parity, and interaction terms; WIM by management system, WIM by parity, lnWIM by management system, and lnWIM by parity were significantly associated (*P *< 0.01) with milk yield at the test day in the type III statistics. Lactation curves for 1st and > 2nd parity cows in conventional and organic farming are presented in Figure 1. The average 8 WIM yield for conventional cows were 24.4, 30.0, and 33.3 kg for 1st, 2nd, and > 2nd lactation, respectively. The corresponding figures for organic cows were 20.4, 26.0, and 29.3 kg.

### Test-day concentrate allocation

**Figure 2:**
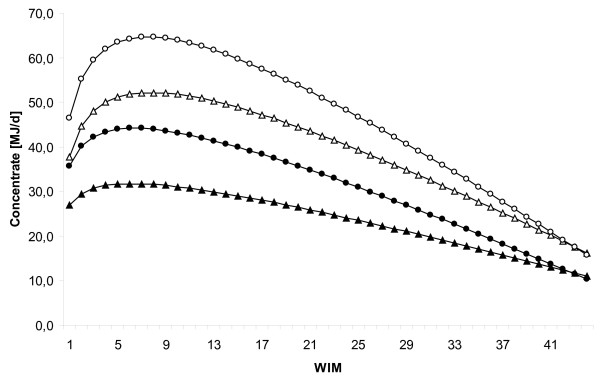
**Lactation curves for concentrate allocation**. Concentrate allocation [MJ/d] by WIM for 1st (organic = black triangle, conventional = white triangle) and > 2nd (organic = black circle, conventional = white circle) parity cows in conventional and organic farming.

All explanatory variables; WIM, lnWIM, management, parity, and interaction terms; WIM by management system, WIM by parity, lnWIM by management system, and lnWIM by parity were associated (*P *< 0.01) with concentrate allocation in the type III statistics. Concentrate allocation curves for 1st and > 2nd parity cows in conventional and organic farming are presented in Figure 2. The average concentrate allocation 8 WIM for conventional cows were 52.2, 61.2, and 64.5 MJ for 1st, 2nd, and > 2nd lactation, respectively. The corresponding figures for organic cows were 31.5, 40.6, and 43.9 MJ.

### Test-day SCC

All explanatory variables; WIM, lnWIM, management, parity, and test-day milk yield, and interaction terms; WIM by management system (*P *= 0.01) and lnWIM by management system (*P *= 0.05) were associated with test-day lnSCC in the type III statistics.

**Figure 3:**
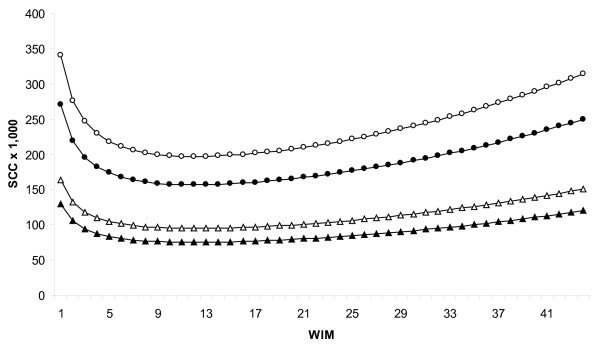
**Lactation curves for SCC**. Test-day somatic cell count (SCC) by WIM for 1st (organic = black triangle, conventional = white triangle) and > 2nd (organic = black circle, conventional = white circle) parity cows in conventional and organic farming.

Curves for back-transformed SCC for 1st and > 2nd parity cows in conventional and organic farming are presented in Figure 3. The back transformed average SCC 8 WIM for conventional cows were 96 900, 135 500, and 202 200 for 1st, 2nd, and > 2nd lactation, respectively. The corresponding figures for organic cows were 77 100, 107 600, and 160 900.

### Herd milk samples

The percentage of quarters dried off due to earlier infection was 2.2% (43/1948) and 1.2% (26/2092) (*P *= 0.02) in organic and conventional management, respectively. Number of quarter milk samples positive for mastitis bacteria is presented in Table 4. There was no difference in the percentage of quarter samples positive for mastitis bacteria between conventional and organic farming. Independent of management, there were more quarter milk samples positive for mastitis bacteria in cows > 2nd parity than in 1st and 2nd parity cows (*P *< 0.01).

**Table 4:** Mastitis bacteria isolated in milk samples

	Conventional		Organic	
Diagnosis	n	%	n	%
Bacteria negative	1746	84.5	1577	82.8
*Staphylococcus aureus*	68	3.3	64	3.4
CNS	167	8.1	200	10.5
*Streptococcus dysgalactiaee*	26	1.3	32	1.7
*Streptococcusococcus uberis*	25	1.2	11	0.6
Other *Streptococcus *spp.	4	0.2	5	0.3
*Escherichia coli*	0	-	3	0.2
*Enterococcus ssp*.	29	1.4	9	0.5
Others^1^	1	0.04	4	0.2

^1 ^*Corynebacterium, Proteus, Archanobachter pyogenes*

Milk samples at quarter level positive for mastitis bacteria in conventional and organic farming at the herd visit (n = 3971).

### Associations between cows with milk samples positive for mastitis bacteria and management system, milk yield, WIM, and parity

When the likelihood of a positive diagnosis for mastitis bacteria (subclinically infected cows) was assessed in separate models for each of the explanatory variables, *P*-values ≤ 0.20 were observed for milk yield in the 4th wk of lactation (*P *< 0.01), WIM (*P *= 0.02) and parity (*P *< 0.01). Multivariate associations between cows with milk samples positive for mastitis bacteria and the explanatory variables are presented in Table 5. In the type III statistics F-test, both parity (*P *= 0.01) and WIM (*P *= 0.04) were associated with the likelihood of at least one quarter positive for mastitis bacteria, whereas milk yield (*P *= 0.07) approached significance. Management system was not associated with bacteriological diagnosis when assessed separately, and hence not included in the model.

**Table 5:** Factors associated with bacteriological positive milk samples.

		β	SE	OR	95% CI for OR	*P*
WIM		0.012	0.006	1.012	1.000, 1.024	0.07
MY		0.023	0.013	1.024	0.998, 1.050	0.04
Parity	1st parity^1^	-	-	-	-	-
	2nd parity	0.023	0.196	1.023	0.697, 1.501	0.91
	> 2nd parity	0.534	0.203	1.707	1.146, 2.540	< 0.01
Intercept		- 1.435	0.355	-	-	< 0.01

^1 ^Categorical variable assigned as baselines

Associations between cows with milk samples positive for mastitis bacteria and explanatory variables; milk yield in the fourth week of lactation (MY), parity and weeks in milk (WIM) at s ampling date at the herd visit in conventional and organic farming (n = 845).

### Samples of cases from clinical mastitis

In the study period, a total of 332 cases with clinical mastitis (229 in conventional and 103 in organic herds) were recorded in 271 cows in 24 conventional and 19 organic herds in the NDHRS-files. With the exception of three cases, all were treated by a veterinarian. In total, there were collected 238 (144 conventional and 94 organic) samples of quarters with clinical mastitis from 177 cows (107 conventional and 70 organic). There were 53 instances where more than one quarter was infected, and treated in the same cow, such that the number of cases of clinical mastitis at the cow level was 185 (112 conventional and 73 organic) from 172 cows (104 conventional and 68 organic). Quarter milk was collected from 48.9% (112/229) of the cases of clinical mastitis cases in conventional herds and from 70.9% (73/103) of the cases in organic herds. Twenty-one conventional herds and 19 organic herds submitted samples from at least 1 cow with clinical mastitis.

The distribution of bacteria isolated from milk samples from quarters with clinical signs of mastitis is presented in Table 6. There was a higher proportion (30.9% versus 18.1%) of clinical mastitis cases where mastitis bacteria were not isolated in organic compared to conventional farming (*P *= 0.02).

**Table 6:** Bacteria isolated from cases of clinical mastitis.

	Conventional		Organic	
Diagnosis	n	%	n	%
No bacteria	26	18.1	29	30.9
Mixed bacteria	2	1.4	1	1.1
*Staphylococcus aureus*	40	27.8	19	20.2
CNS	5	3.5	1	1.1
*Streptococcus dysgalactiaee*	16	11.1	16	17.0
*Streptococcus uberis*	8	5.6	2	2.1
Other *Streptococcus *spp.	3	2.1	2	2.1
*Escherichia coli*	34	23.6	18	19.1
*Enterococcus *spp.	1	0.7	0	-
Others^1^	9	6.3	6	6.4

^1 ^*Proteus, Klebsiella, Archanobacter pyogenes, Pasturella, Serratia*, yeast, combinations of *S. aureus *and *Streptococcus dysgalactiae*.

Distribution of bacteria isolated from milk samples from quarters with sign of clinical mastitis (changes in milk secret or symptoms from quarter) in conventional and organic farming (n = 238, from 177 lactations 173 animals).

### Antibiotic resistance in milk samples from the herd visit and samples from clinical mastitis

Of the staphylococci isolated from milk samples of subclinically infected quarters collected during the routine visits to the conventional herds, 81 of the 167 isolates (48.5%) of coagulase-negative staphylococci (CNS) and 6 of the 68 isolates (8.8%) of *S. aureus *were resistant to penicillin. The corresponding findings in the organic herds were for CNS 93 penicillin-resistant isolates out of 200 (46.5%) and for *S. aureus *nine penicillin-resistant isolates out of 64 (14.0%).

Of the isolates from quarters with clinical mastitis, resistance to penicillin was not found among the 59 *S. aureus *from conventional and organic herds. Whereas one out of one of the CNS isolates from the organic herds, and three out of five of the CNS isolates from conventional herds were penicillin-resistant.

## Discussion

Previously, there have been separate Norwegian field studies comparing reproductive performance [4] udder health [5], and herd health and management [13] in conventional and organic dairying. The present study agrees with previous findings of higher parity distribution and lower milk yield in organic cows [4,5]. However, the present study does not support the reported findings of impaired reproductive performance in organic herds [4] or the statement of no differences in SCC [5] between the management systems.

Roesch et al. reported that lower milk yields in organic farms were due to individual animal and farm level factors; such as udder health, breed, nutrition and management [8]. In this study conventional herds were selected to the organic herds on the basis of geographical location, housing system and herd size. Consequently, these potential confounders were unlikely to have influenced the findings of the present study. Since the cows included in the study were all Norwegian Reds, breed is not a potential confounder either. All organic herds included in the study converted form 1997 to 2003. During this period the government provided an economic incentive for conversion to organic farming. It may have been this, rather than the idealism of farming in a sustainable manner, that stimulated conversion, as described in Switzerland [33]. The governmental payment could have affected management decisions more than the holistic view of organic production in the present study, which could be interpolated to other Norwegian organic farmers converting to organic production after 1997.

Veterinary treatment is shown to be influenced of cow and herd characteristics [34,35]. Thus, the lower number of cases treated for reproductive disorders and clinical mastitis reported to NDHRS could be due to the limited use of antibiotics in organic farming, such that veterinarian treatments are not performed and the cases are not reported to the NDHRS-files.

Some of the milk samples from the herd visit were stored frozen until bacteriological examination. Schukken et al. reported that freezing 4 to 16 weeks after collection decreased the number of samples with cultures of *E. coli*, increased cultures of CNS whereas freezing had no effect on streptococci and *S. aureus *[36]. Dinsmore et al. found an overall higher positive culture rate in frizzed milk samples, but freezing did not affect the positive culture rate of any individual bacterial species [37]. No milk samples were stored frozen for more than 13 d. Thus, we feel confident that the sensitivity of diagnosed bacteria was not affected by freezing of some samples.

The organic cows were fed less concentrate than conventional cows. Reksen et al. reported that the energy provided by concentrate for most organic herds was 20%, because a maximum of 20% of the feed could be non-organic in origin and production of organic grain was limited[4]. The percentage of non-organic feed allowed to use on organic farms has gradually been reduced. Currently, 60% of the energy fed on daily basis should be roughage [3]. Hence, today it is possible to feed a higher amount of concentrate than the limit of 20% ten years ago. In 2007, 39.4% of the feed fed to Norwegian cows per cow-year was concentrate [38]. Valle et al. reported that the percentage concentrate fed was 39.3% and 27.4% for conventional and organic herds, respectively [13]. In the present study, the average annual forage: concentrate ratio was 63:37 in conventional herds and 75:25 in organic herds. Previously, it has been described that Norwegian Red cows are able to maintain ovarian activity by decreasing milk yield when the forage to concentrate ratio was 75:25 or lower [39]. Fall et al. reported that organic cows did not mobilize more body tissue than conventional cows, and that the organic cows adjusted the milk production according to feed intake [6]. It could be that the organic cows decreased milk yield according to feed intake and hence met the negative energy balance post partum with lower milk yield rather than impaired reproductive performance since milk yield was not associated with any of the fertility measurement investigated in the present study.

Natural mating as first service was more commonly used in organic than conventional farming, which agrees with the findings reported by Reksen et al. [4]. The interval to first AI was shorter for conventional cows, whereas there was no difference in calving interval, days open, and interval to last AI between the management systems. Reksen et al. and Valle et al. reported no difference in these reproduction measures except for a reduction in the number of days open for organic cows [4,13] whereas Löf et al. reported shorter calving intervals and shorter interval to first and last AI in organic cows [11]. Nauta et al. found extended calving interval in cows managed on farms that converted between 1990 and 2003 [7]. Cows in free stall herds had shorter interval to first AI, last AI and calving interval in the present study. Valde et al. reported higher fertility status index in free stall herds compared to tie stall herds [40]. When cows only presented for natural mating were investigated, organic cows had shorter calving interval and fewer days open than conventional cows which could be explained by more complete systems with natural breeding as first service compared to conventional farming. There were no difference between season for AI and the two types of farming which contrasts with the findings of Reksen et al. where 52% of the organic cows and 36% of the conventional cows were bred during the summer [4].

Although adjusted for parity and milk yield, SCC was lower in organic cows during the whole lactation period compared to conventional cows which contrasts with the findings that no difference in SCC existed between the management systems [5,9,15]. A Danish and a Dutch study reported lower milk yield in long-standing organic farms compared to later conversion and conventional farms, but the studies were conflicting regarding to SCC [7,41]. A Swedish study found lower milk yield and lower proportion of cows with high SCC in organic farming [42], whereas Valle et al. [13] found no differences in bulk milk SCC and higher culling rate in conventional herds. In the present study, the culling rate was slightly higher in the conventional herds which should give the opportunity to cull cows with high SCC and hence potential to improve the udder health.

The percentage of dry quarters at the herd visits was higher in organic compared to conventional farming which could have given lower measurements of SCC in organic herds. Valle et al. reported a lower proportion of produced milk delivered to the dairy factory from organic herds [13]. Hamilton et al. reported that organic farmers were more unlikely to use veterinary treatment in cases of clinical mastitis [35]. In the present study, four organic farmers always preferred veterinary treatment in cases of clinical mastitis, whereas 12 conventional farmers always called the veterinarian. In a previous study, it was reported that conventional farmers on average called the veterinarian in 4.7 out of 10 mild mastitis cases, whereas the corresponding figure was 2.0 for organic farmers [13]. In 2007, 16 of the 24 organic farms had reported data about (veterinary) treatment in cases of mastitis to the NDHRS-files, whereas 22 of 25 conventional farms had reported cases of mastitis. Milk was sampled in 48.9% of the clinical mastitis cases treated in conventional herds whereas the corresponding figure was 70.9% in organic herds. The milk samples from cases with clinical mastitis were sampled from cows in 21 conventional herds and 19 organic herds. The number of samples submitted from farms varied considerably, especially for the conventional farmers (1 to 23). This variation could be due to differing levels of motivation to improve the udder health in each herd.

The odds for a cow to be subclinically infected in at least one quarter were not associated with management, but parity and milk yield. There was no difference between organic and conventional herds with respect to bacteriologically positive milk samples at the herd visit. Coagulase-negative staphylococci were the bacteria most frequently isolated from quarters in both conventional (8.1%) and organic (10.5%) cows. This differs considerably from the reported frequency of 3.3% in a nationwide random assignment survey [43]. The percentage of milk samples positive for *Streptococcus dysgalactiae *at the herd visits was similar in the two management systems, and corresponds well with the 1.2% of cows previously reported to be infected national [43]. The proportion of *S. aureus *was similar in organic and conventional herds (3.3 and 3.4%), but lower than previous reports of 8.2% [43]. For *Streptococcus uberis*, the percentage (0.6%) of organic quarters infected corresponds with the report of Østerås et al. [43]. However the infection rate in conventional herds was twice as high (1.2%) as the earlier report. The deviation of the proportion of mastitis bacteria isolated in the present study compared to the study conducted by Østerås et al. [43] could be due to the design of the studies. In the present study, all cows in lactation in the herd were sampled, whereas the other study sampled every fifth cow from every 50th herd during each quarter of the year [43]. Hence environmental factors in the specific herd in the present study would have larger impact on the proportion of infected quarters compared to the other study.

In the clinical mastitis cases, there were a higher proportion of bacteriological negative milk samples from organic farms (30.9% versus 18.1%). The reason for this remains unknown, but it could be possible that some of this quarters originally were infected by *E. coli *which was not present at the time of milk sampling or it could be a result of clustering of samples in specific farms with specific pathogens. For the clinical mastitis cases in the present study only percentages of quarters infected in organic and conventional farming were presented. Vaarst and Enevoldsen reported that bacteriological negative mastitis showed strong similarities with clinical coliform mastitis, and that 20% of the cases were bacteriological negative in a study of manifestation of clinical mastitis in Danish organic herds [44]. In another Norwegian study, 12.5% of the cases of clinical mastitis were bacteriological negative [45]. *Staphylococcus aureus *was the bacteria most frequently isolated from quarter samples with signs of clinical mastitis in both conventional (27.8%) and organic herds (20.2%), which is lower than previous reported in heifers (pre partum to 14 d post partum) of 44.3% [46]. Whist et al. reported *S. aureus *to be isolated in 47.4% of the cases with clinical mastitis in problem herds [45]. In the present study, *E. coli *was the second most frequent reason for clinical mastitis in both conventional (23.6%) and organic (19.1%) cows. The proportions in this study are higher than previous reported of 6.4% [46] and 10.7% [45]. The frequency of *Streptococcus dysgalactiae *in organic farming (17.0%) was close to previous studies with 18.2% [46] and 22.5% [45]. In conventional farming, the percentage of *Streptococcus dysgalactiae *was only 11.1%. The reason for such low proportion of *Streptococcus dysgalactiae *in conventional farming could be due to higher motivation to improve udder health and more use of dry cow therapy initiated by veterinarian than organic herds. The use of dry cow therapy was not investigated in the present study.

Cases selected for antibiotic treatment are assessed by the veterinarian before treatment. Benzyl penicillin and dihydrostreptomycin are the most common antibiotics used for intramammary treatment [40,45]. Penicillin resistance against *S. aureus *was not found in the cases of clinical mastitis. At the herd visit, penicillin resistance against *S. aureus *was found in 8.8% of the subclinically infected quarters in conventional farming and in 14.1% of subclinically infected quarters in organic farming. Østerås et al. reported 11.4% resistance against penicillin G for *S. aureus *in subclinically infected quarters [43]. Other studies investigating antibiotic resistance between the two management systems reported no difference in penicillin resistance between the two farming types [16,47]. Tikofsky et al. found that *S. aureus *in US organic farms were more susceptible to antibiotics than in conventional farms, but also that *S. aureus *isolates in general showed good susceptibility [20]. Pol and Ruegg reported that *S. aureus *isolates are more likely to be resistant against penicillin in conventional herds [17]. Since the choice of antibiotics and cases for treatment are very different in US and Norway, comparisons between the countries are difficult. A study that investigated antimicrobial susceptibility of *S. aureus *in bulk tank milk in organic and conventional farms in Denmark and US, reported small differences between organic and conventional farms in each country, but large differences between the respective agricultural systems [21]. Penicillin resistance against CNS isolated from subclinically infected quarters was 48.5% in conventional and 46.5% in organic herds. Østerås et al. reported that 36.1% of the CNS isolates from subclinically infected quarters were penicillin resistant and that the resistance was highest during late indoor season (April-May) [43]. This could explain the higher percentage of penicillin resistance CNS in the present study, because most of the herd visits took place in the spring and early summer (late March to late May) in both conventional and organic herds.

## Conclusion

There were no differences in the interval to first AI, interval to last AI or calving interval between organic and conventional farming. The cows were older in organic farming. Conventional cows yielded more, had higher SCC, and received more concentrates than organic cows. Higher level of concentrate fed to organic cows in recent years is probably an important factor for higher reproductive efficiency in organic cows than ten years ago.

## Competing interests

The authors declare that they have no competing interests.

## Authors' contributions

RTG contributed to design, planning and administration of the study, performed the herd visits, collection, analysis, and interpretation of data, main author of the manuscript. SW contributed to study concept and design, analysis and interpretation of data, critical revision of the manuscript for important intellectual content. SS contributed in the udder health part and analysis of milk samples, reviewing the manuscript with special emphasis on the udder health part. BIFH contributed in the selection of organic herds, ensured correct information about organic farming and regulations, and production of the manuscript. OØ contributed in the planning of data needed for epidemiological analysis, collection of data from the Norwegian Dairy Herd Recording System, input on epidemiological and statistical approach in the manuscript. OR contributed in design, planning, analysis and interpretation of data, critical revision and production of the manuscript.

All authors read and approved the final manuscript.
